# NSAID use may decrease serum Klotho levels

**DOI:** 10.3389/fendo.2025.1531325

**Published:** 2025-04-16

**Authors:** Jingchao Yan, Hong Sun, Xiu Xin, Taomin Huang

**Affiliations:** Department of Pharmacy, Eye & ENT Hospital, Fudan University, Shanghai, China

**Keywords:** NSAIDs, cyclooxygenase, inflammation, aging, α-Klotho (Klotho)

## Abstract

**Objective:**

To validate the hypothesis proposed by previous studies, which suggests that NSAID use may elevate Klotho levels.

**Method:**

We conducted a cross-sectional study involving 11,626 adults from the National Health and Nutrition Examination Survey (NHANES) 2007-2016. Multivariable linear regression and propensity score analysis were employed to evaluate the association between NSAID use and serum Klotho levels. Additionally, subgroup analyses were performed to assess the consistency of this relationship across various subgroups.

**Results:**

Multivariable linear regression analysis demonstrated that NSAID use was negatively correlated with serum Klotho levels (β = -25.48 [95% CI: -42.00, -8.96], p = 0.003). Additionally, sensitivity analysis results were consistent with the primary analysis. Subgroup analyses did not reveal any statistically significant interactions.

**Conclusion:**

Contrary to previous speculations, the use of NSAIDs is associated with a decrease in serum Klotho levels.

## Introduction

The α-Klotho protein, which is encoded by the KL gene, was first identified in 1997 (1, 2). The name α-Klotho is derived from Clotho, the Greek goddess who spins the thread of life, symbolizing its role in the aging process (3). The α-Klotho protein exists in three forms: a full-length transmembrane form, and soluble and secreted isoforms. The membrane-bound α-Klotho is cleaved to produce the soluble form, while the secreted isoform is generated through selective RNA splicing. The soluble and secreted forms share similar structures and together constitute the circulating serum α-Klotho (hereafter referred to as Klotho) (4–6).

Klotho has been implicated in a range of age-related conditions, including Alzheimer’s disease (7, 8), cerebrovascular diseases (9), and Parkinson’s disease (10). Furthermore, research suggests that Klotho plays a protective role in preserving high-frequency hearing (6), mitigating the elevation of blood pressure induced by high salt intake (11), and influencing the development of colorectal cancer (12), cancer metastasis (13), and metabolic disorders (14).

The role of Klotho in longevity and disease prevention has garnered significant interest, prompting researchers to investigate the factors that regulate its expression (15). Studies have shown that inflammation can reduce Klotho expression, leading to speculation that anti-inflammatory drugs may help maintain or even increase Klotho levels (15, 16). However, this hypothesis requires further validation through more precise studies. To test this, we conducted the present study.

## Materials and methods

### Data sources and study population

The National Health and Nutrition Examination Survey (NHANES) is a nationwide study carried out by the National Center for Health Statistics (NCHS) to provide a representative sampling of the American population’s health and nutrition status (17). The Centers for Disease Control and Prevention (CDC) oversees the survey, employing a complex multistage sampling approach to select a nationally representative sample biennially.

The study was approved by the NCHS Research Ethics Review Board (https://www.cdc.gov/nchs/nhanes/irba98.htm), with IRB/ERB Protocol Numbers #2005-06 and #2011-17. Participants provided written informed consent in accordance with the Declaration of Helsinki. The data are publicly available through the CDC website: https://www.cdc.gov/nchs/nhanes/ (last accessed on March 1, 2023). We extracted demographic information, survey responses, and laboratory test results, including serum creatinine (Scr) and Serum Klotho concentrations, from the NHANES datasets collected between 2007 and 2016. These years covered all instances in which Klotho concentrations were measured. The sample size was determined based on the available data, with no prior power calculations. This study adhered to the STROBE (Strengthening the Reporting of Observational Studies in Epidemiology) guidelines for reporting.

### Exposure

NSAID usage was obtained from the NHANES Prescription Medications Questions. Participants were asked, “Have you taken or used any prescription medications in the past month?” Those who used NSAIDs were categorized as the exposed group, while those who did not were classified as the control group. In this study, NSAIDs included 17 different drug types, such as acetaminophen, ibuprofen, and aspirin. A comprehensive list of these drugs is provided in **Supplementary Table S1**.

### Serum Klotho protein

Participants followed an overnight fasting protocol in accordance with established guidelines. Blood samples were subsequently collected and immediately stored at -80°C. These samples were first transported on dry ice to the mobile examination center and then transferred to the Northwest Lipid Metabolism and Diabetes Research Laboratories at the University of Washington for analysis. Serum Klotho levels were measured using a commercial ELISA kit from IBL International, Japan. To ensure quality control, each ELISA plate included duplicate samples with either low or high Klotho concentrations, and tests were repeated if any results deviated by more than two standard deviations from the expected values. The assay’s detection limit was 6 pg/mL, and since all samples exceeded this threshold, no imputation was required. Additional details on laboratory methods and quality assurance procedures can be found at: https://wwwn.cdc.gov/Nchs/Nhanes/2007-2008/SSKL_E.htm.

### Covariates

Potential covariates were assessed based on both biological plausibility and existing literature. These factors included demographic variables (such as age, sex, race/ethnicity, education level, poverty-income ratio [PIR], and marital status), health-related behaviors (such as smoking, alcohol consumption, and physical activity), and clinical indicators (including body mass index [BMI], diabetes [DM], hypertension, and estimated glomerular filtration rate [eGFR]).

Age was considered a continuous variable. The participants’ self-reported race/ethnicity was grouped into five categories: Mexican American, other Hispanics, non-Hispanic white, non-Hispanic black, and other racial groups. Educational level was divided into two categories: individuals without a high school diploma and those who had completed high school or obtained a higher level of education. Marital status was classified into two groups: those who were married or living with a partner, and those who were living alone, including individuals who were widowed, divorced, or separated. The Poverty Income Ratio (PIR), which represents the ratio of family income to the federal poverty line, ranged from 0 to 5. Smoking behavior was categorized based on established definitions, with participants classified as never smokers (having smoked fewer than 100 cigarettes), current smokers, or former smokers (having quit after smoking more than 100 cigarettes). Alcohol consumption was assessed with the question, “In the past year, have you consumed at least 12 drinks of any alcoholic beverage?” Those who responded affirmatively were classified as alcohol users. During the physical examination, height and weight were measured, and BMI was subsequently calculated. Physical activity was classified into three intensity levels: sedentary, moderate, and vigorous. Moderate physical activity was defined as at least 10 minutes of movement in the past 30 days that resulted in light sweating or a mild to moderate increase in breathing or heart rate, while vigorous activity required a similar duration of movement but led to heavy sweating or a substantial rise in breathing or heart rate. The diagnosis of DM was based on the criteria established by the American Diabetes Association (18). Participants were categorized as having DM if they fulfilled any of the following conditions: (1) self-reported diabetes diagnosis by a healthcare provider; (2) use of either oral hypoglycemic agents or insulin; or (3) a fasting plasma glucose level ≥ 126 mg/dL, a 75 g oral glucose tolerance test (OGTT) result ≥ 200 mg/dL, or a hemoglobin A1c (HbA1c) level ≥ 6.5%. Hypertension was defined as having a systolic blood pressure ≥ 140 mm Hg and/or a diastolic blood pressure ≥ 90 mm Hg, or as a self-reported diagnosis from a healthcare provider. Chronic kidney disease (CKD) was diagnosed when the eGFR was below 60 mL/min/1.73 m². The eGFR was calculated using the 2021 Chronic Kidney Disease Epidemiology Collaboration (CKD-EPI) equation: GFR = 142×min [Scr/κ, 1] ^α^ × max [Scr/κ, 1]^-1.200^ × 0.9938^Age^ × 1.012 [if female]. In this formula, κ was set to 0.7 for females and 0.9 for males, α was -0.241 for females and -0.302 for males, while “min” and “max” refer to the minimum and maximum of Scr/κ or 1, respectively (19). Further details on these variables can be accessed through the NHANES website at https://www.cdc.gov/nchs/nhanes/index.htm.

### Statistical analysis

We conducted a descriptive analysis of all participants. Categorical variables are presented as counts and percentages, while continuous variables are summarized as either the mean and standard deviation (SD) for normally distributed data or the median and interquartile range for skewed distributions. To compare categorical variables and continuous variables with different distributions, we applied the chi-square test, one-way ANOVA, and the Kruskal-Wallis test, respectively.

Multivariable linear regression models were applied to investigate the relationship between NSAID use and serum Klotho levels. Additionally, propensity score matching (PSM) and propensity score adjustment were used to assess the impact of NSAID use on serum Klotho levels, while minimizing potential biases related to NSAID allocation and confounding factors (20). A 1:1 nearest neighbor matching algorithm was implemented with a caliper width set at 0.2. The following covariates were included in the propensity score model: age, sex, race, education level, marital status, BMI, PIR, smoking habits, alcohol consumption, physical activity, hypertension, DM, and eGFR. To evaluate the balance achieved by PSM, the standardized mean difference (SMD) was calculated, with an SMD threshold of 0.1 deemed acceptable. Furthermore, we performed a multivariate linear regression analysis to compare serum Klotho levels between populations using NSAIDs, those using other medications, and individuals not using any medication.

Statistical analyses were performed using the R 4.3.2 software package (http://www.R-project.org, The R Foundation) and the Free Software Foundation’s statistics software version 1.7.1. A significance threshold of p < 0.05 was set. The sample size was determined based on the available data, without prior power analysis.

## Results

### Characteristics of the participants

**Figure 1:**
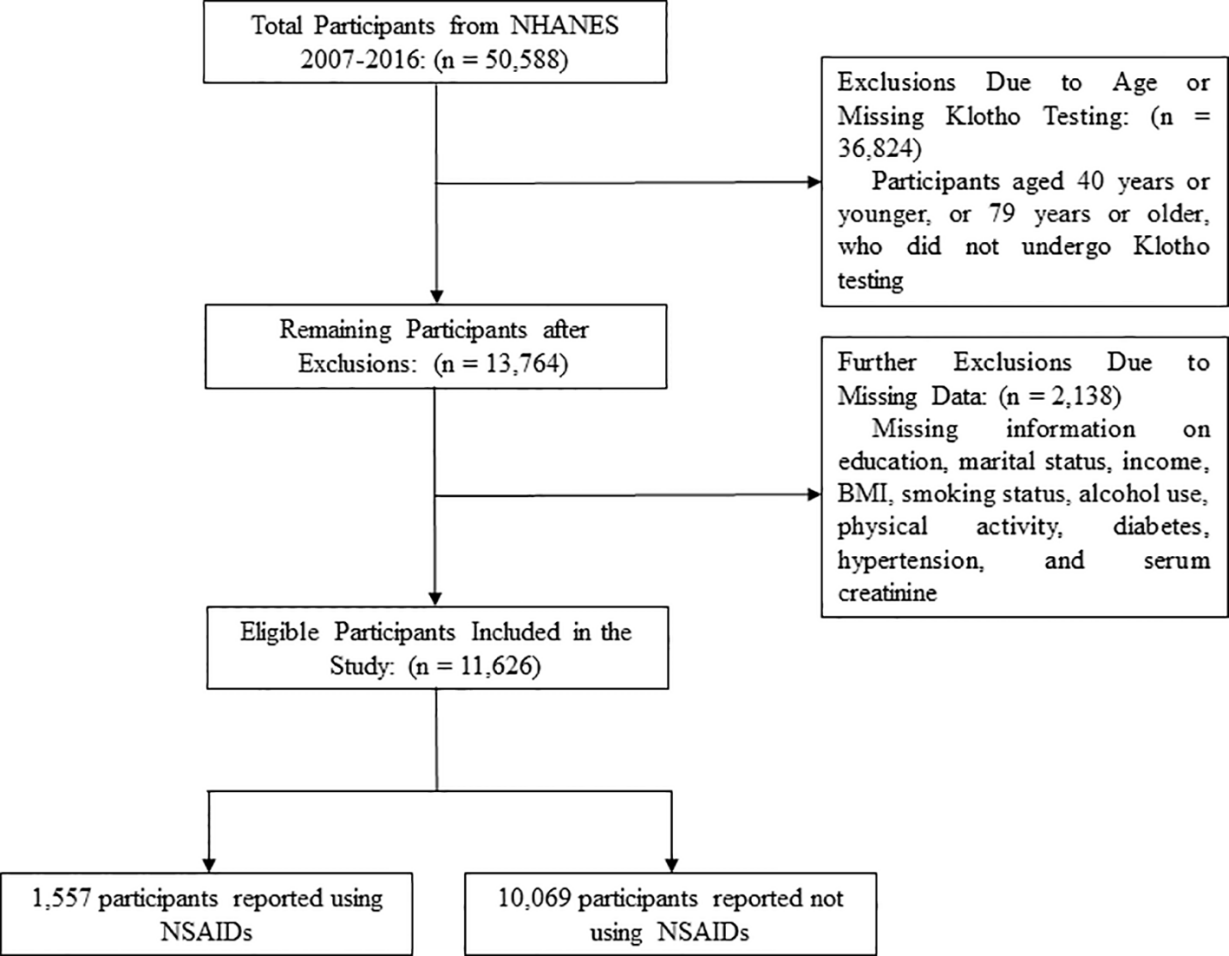
Participant enrollment flowchart.

After screening the original dataset, a total of 11,626 participants, aged 40 to 79 years, were included in this study (the screening process is detailed in **Figure 1**). Compared to non-users, NSAID users were older, more likely to be female, had lower levels of education and income, were more likely to live alone, engaged in physical activity less frequently, and had a higher prevalence of smoking, alcohol consumption, hypertension, DM, and a lower eGFR (see **Table 1**).

**Table 1:** Baseline characteristics of study participants.

Variables	All participants (n =11626)	NSAIDs usage		*p*-value
		No (n =10069)	Yes (n =1557)	
Age (years), Mean ± SD	57.8 ± 10.8	57.5 ± 10.8	59.7 ± 10.5	< 0.001
Male, n (%)	5699 (49.0)	5037 (50.0)	662 (42.5)	< 0.001
Race/ethnicity, n (%)				< 0.001
Mexican American	1774 (15.3)	1536 (15.3)	238 (15.3)	
Other Hispanic	1251 (10.8)	1096 (10.9)	155 (10.0)	
Non-Hispanic White	5282 (45.4)	4537 (45.1)	745 (47.8)	
Non-Hispanic Black	2296 (19.7)	1969 (19.6)	327 (21.0)	
Other Race	1023 (8.8)	931 (9.2)	92 (5.9)	
Level of education, n (%)				< 0.001
Did not graduate from high school	3089 (26.6)	2614 (26.0)	475 (30.5)	
high school level or above	8537 (73.4)	7455 (74.0)	1082 (69.5)	
Marital status, n (%)				< 0.001
Living alone	4117 (35.4)	3462 (34.4)	655 (42.1)	
Married or living with a partner	7509 (64.6)	6607 (65.6)	902 (57.9)	
BMI, kg/m^2^, Mean ± SD	29.8 ± 6.8	29.6 ± 6.5	31.7 ± 8.0	< 0.001
PIR, (IQR)	2.2 (1.2, 4.4)	2.4 (1.2, 4.6)	1.6 (0.9, 3.3)	< 0.001
Smoking status, n (%)				< 0.001
Never	5861 (50.4)	5216 (51.8)	645 (41.4)	
Current	2295 (19.7)	1900 (18.9)	395 (25.4)	
Past	3470 (29.8)	2953 (29.3)	517 (33.2)	
Alcohol status, n (%)				0.347
No	8278 (71.2)	7185 (71.4)	1093 (70.2)	
Yes	3348 (28.8)	2884 (28.6)	464 (29.8)	
Physical activity, n (%)				< 0.001
Sedentary	4224 (36.3)	3521 (35.0)	703 (45.2)	
Moderate	3997 (34.4)	3498 (34.7)	499 (32.0)	
Vigorous	3405 (29.3)	3050 (30.3)	355 (22.8)	
Hypertension, n (%)				< 0.001
No	5164 (44.4)	4673 (46.4)	491 (31.5)	
Yes	6462 (55.6)	5396 (53.6)	1066 (68.5)	
Diabetes, n (%)				< 0.001
No	8710 (74.9)	7654 (76.0)	1056 (67.8)	
Yes	2916 (25.1)	2415 (24.0)	501 (32.2)	
eGFR, (mL/min/1.73 m²)	87.7 ± 19.6	88.1 ± 19.5	84.9 ± 20.4	< 0.001
Serum Klotho (pg/mL), (IQR)	801.4 (654.3, 991.2)	806.3 (658.9, 996.3)	768.4 (612.4, 955.3)	< 0.001

BMI, body mass index; PIR, poverty income ratio; eGFR, estimated glomerular filtration rate.

### Results of the multivariate linear regression analysis of the effect of NSAID use on serum Klotho levels

To minimize confounding effects, we constructed four stepwise-adjusted generalized linear regression models to analyze the independent effects of NSAID use on serum Klotho levels. The effect sizes (β) and 95% confidence intervals (CIs) are presented in **Table 2**. In the unadjusted model, NSAID use was negatively correlated with serum Klotho levels (β = -35.05 [95% CI -51.56, -18.53]) compared to participants who did not use NSAIDs. After adjusting for confounding factors, this negative association remained consistent.

**Table 2:** Multivariate linear regression analysis of the effect of NSAIDs use on serum Klotho levels.

NSAIDs usage	Model 1		Model 2		Model 3		Model 4	
	β (95% CI)	*p*-value	β (95% CI)	*p*-value	β (95% CI)	*p*-value	β (95% CI)	*p*-value
No	Reference		Reference		Reference		Reference	
Yes	-35.05 (-51.56, -18.53)	<0.001	-33.73 (-50.17, -17.30)	<0.001	-30.49 (-47.10, -13.88)	<0.001	-25.48 (-42.00, -8.96)	0.003

β is the effect size (pg/mL) of the change in serum klotho level, and the 95% CI indicates the 95% confidence interval.

Model 1 Unadjusted model.

Model 2 adjusted for age + sex + race.

Model 3 adjusted for Model 2+ education + marital status + PIR+ BMI.

Model 4 adjusted for Model 3 + smoking status + drinking status + physical activity + diabetes + hypertension + eGFR.

Abbreviations as Described in **Table 1**.

### Subgroup analysis

**Figure 2:**
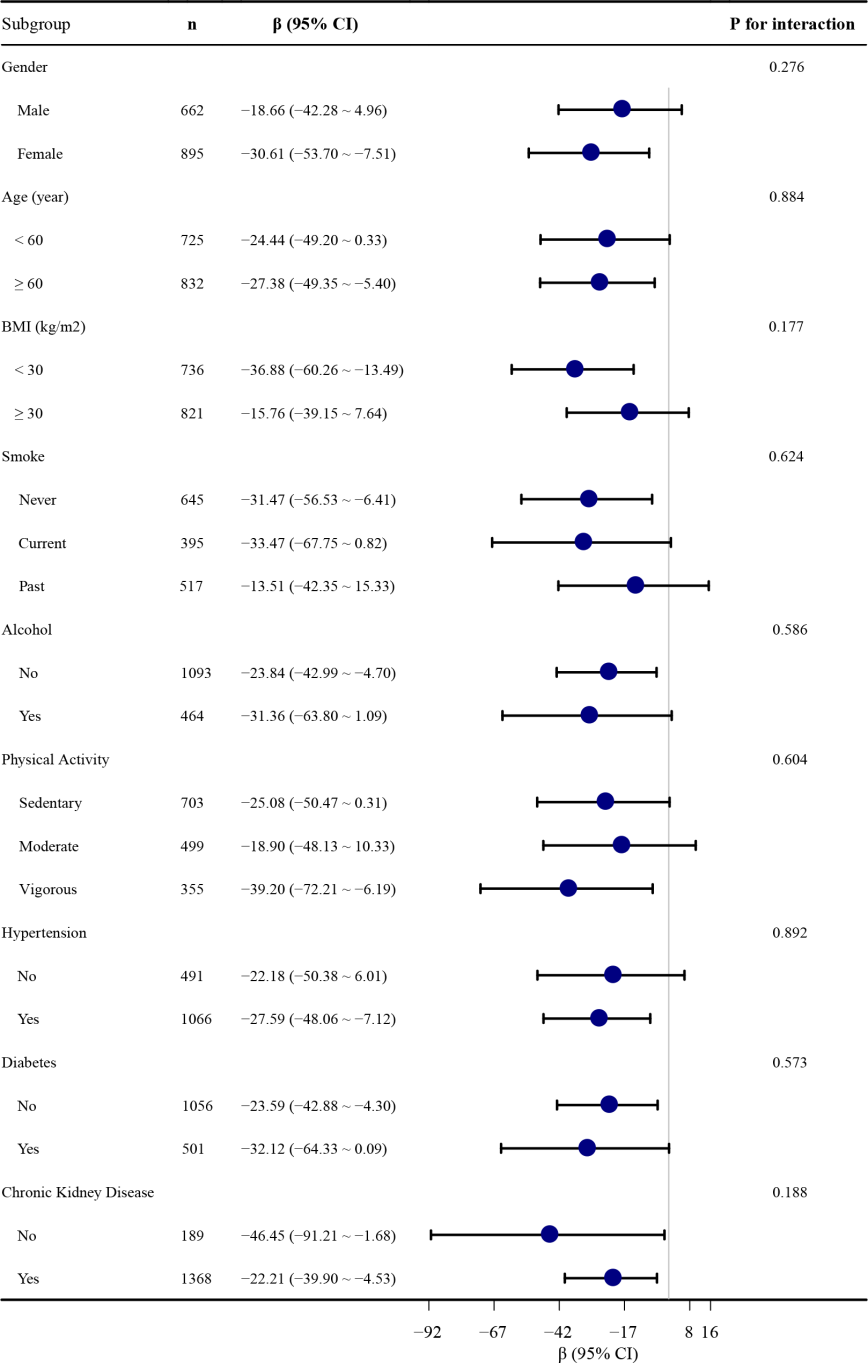
Association between NSAID use and serum S-Klotho levels stratified by baseline characteristics. Each stratification is adjusted for all covariates in **Table 2**, Model 4, except for the stratification itself.

To further explore these relationships, we conducted stratified analyses based on baseline characteristics to assess the consistency of the association between NSAID use and serum Klotho levels (see **Figure 2**). No statistically significant interactions were observed in these subgroup analyses examining effect modification.

### Sensitivity analyses

To minimize potential biases related to NSAID allocation and confounding factors, we employed PSM and propensity scores adjustment to assess the impact of NSAID use on serum Klotho levels. PSM resulted in 1,551 well-balanced pairs, with baseline variables comparable across groups (**Supplementary Table S2**). Additionally, we performed a multivariate linear regression analysis to compare serum Klotho levels among individuals using NSAIDs, those using other medications, and those not using any medications. The results were consistent with the primary analysis, indicating that NSAID use was negatively correlated with serum Klotho levels (see **Table 3**).

**Table 3:** Associations between NSAID use and serum Klotho levels: crude, multivariable linear regression, and propensity score analysis.

Analysis	β (95% CI)	*p*-value
Multivariable linear regression model		
NSAIDs Usage vs. Use of Other Medications **^a^** (1557/6578)	-28.66 (-46.11, -11.21)	0.001
NSAIDs Usage vs. Non-Use of Any Medications **^a^** (1557/3491)	-24.79 (-45.40, -4.18)	0.018
Multivariable analysis		
With matching **^b^**	-23.75 (-45.12, -2.37)	0.029
Adjusted for propensity score **^c^**	-26.29 (-43.15, -9.43)	0.002

**^a^** Shown is the effect size from the multivariable linear regression model, adjusted for all covariates in **Table 2**, Model 4.

**^b^** Shown is the effect size from the multivariable linear regression model, adjusted for the same covariates as in **Table 2**, Model 4, with matching based on the propensity score.

**^c^** Shown is the effect size from the multivariable linear regression model, adjusted for the same covariates as in **Table 2**, Model 4, with additional adjustment for the propensity score.

## Discussion

In this study, we investigated the relationship between NSAID use and blood Klotho levels. Our findings revealed a significant negative correlation between NSAID use and blood Klotho levels. We employed multiple analytical methods to validate the results, and both multivariable linear regression and propensity score analyses confirmed the robustness of the findings.

To the best of our knowledge, this is the first study to examine the relationship between NSAID use and blood Klotho levels in participants, thereby challenging previous hypotheses that the use of anti-inflammatory drugs may help maintain or even elevate Klotho expression (15). While existing research has explored the role of inflammation in the reduction of Klotho (16, 21, 22), it was previously hypothesized that anti-inflammatory drugs might help maintain or even increase Klotho expression. However, contrary to this speculation, our study found that NSAID use is associated with a decrease in blood Klotho levels.

NSAIDs exert their effects by inhibiting cyclooxygenase (COX) activity (23), an enzyme present in various tissues, including the kidneys, where it plays a crucial role in maintaining essential physiological functions such as renal blood flow (24, 25). Due to COX inhibition, NSAIDs decrease the synthesis of prostaglandins (26–28), which are essential for regulating renal blood flow (RBF) and glomerular filtration rate (GFR). This inhibition may reduce the production of local vasodilators in the kidneys, leading to renal vasoconstriction and a subsequent decline in RBF. (29). This could, in turn, lower Klotho levels. Additionally, NSAIDs may disrupt fluid and electrolyte balance and elevate blood pressure (30, 31), both of which can further affect renal blood flow and serum Klotho levels.

We hypothesize that the reduction in serum Klotho levels associated with NSAID use may be related to the following mechanisms: Klotho protein is primarily expressed in the kidneys, and kidney disease can disrupt renal Klotho expression. Studies have shown that a decrease in kidney mass is associated with reduced Klotho levels (32). Renal injury, a known adverse effect of NSAID use, is linked to both acute and chronic kidney damage (33–35). Therefore, NSAIDs may contribute to a reduction in Klotho levels by exacerbating kidney injury (32).

NSAIDs exert their effects by inhibiting cyclooxygenase (COX) activity, an enzyme expressed in various tissues, including the kidneys, where it is crucial for maintaining fundamental physiological functions such as renal blood flow by inhibiting COX, NSAIDs reduce the synthesis of prostaglandins, which play a key role in regulating renal blood flow (RBF) and glomerular filtration rate (GFR).

In summary, NSAID medications may influence blood Klotho levels through multiple mechanisms, including reductions in renal blood flow and glomerular filtration rate, increased renal injury, and disruptions in fluid and electrolyte balance and blood pressure. These mechanisms may act independently or synergistically, collectively contributing to decreased blood Klotho levels. This observational study was conducted in a human population. It is recommended that future animal experiments be conducted to validate these findings and further explore the underlying mechanisms.

## Limitations

This study has several limitations. Firstly, due to its cross-sectional design, we are unable to establish a causal relationship between NSAID use and serum Klotho levels, and there may be unmeasured confounding factors. We suggest that future research consider these potential confounders, such as specific dietary habits and the use of other medications. Additionally, serum Klotho levels were measured at a single time point in the NHANES dataset, and we lacked information on potential fluctuations over time. Various factors could influence Klotho levels, leading to abnormally high or low measurements at the time of assessment. Future research should explore the temporal variability of serum Klotho concentrations. Furthermore, different classes of NSAIDs, with their distinct chemical structures and mechanisms of action, may have differential effects on blood Klotho levels. Subsequent studies will investigate the individual impacts of NSAIDs with varying mechanisms on Klotho levels.

## Conclusion

This study provides evidence suggesting an association between NSAID use and lower serum Klotho levels, which contrasts with prior speculations that NSAIDs might elevate Klotho levels. Our findings, based on a large cohort of 11,626 adults from the NHANES dataset, were consistent across various analytical methods, including multivariable linear regression and propensity score analysis. Due to NSAID diversity, individual variability, and underlying conditions, these results should be interpreted cautiously. Further studies are needed to confirm the findings and establish causal links.

## Data Availability

Publicly available datasets are available online for this study. The repository/repositories name and accession numbers are available online at http://www.cdc.gov/nchs/nhanes.htm (accessed on March 1, 2023). Further inquiries can be directed to the corresponding authors.
