# Supplementary material for: NSAID use may decrease serum Klotho levels
Source: Front Endocrinol (Lausanne). 2025 Apr 16;16:1531325. doi: 10.3389/fendo.2025.1531325 (PMC12040623; doi:10.3389/fendo.2025.1531325)
Supplement: Supplementary file 1 [file Table1.docx]

Table S1. List of Nonsteroidal Anti-Inflammatory Drugs (NSAIDs) Included in the Study.

| Serial Number | Generic Name of the Medicine |
| --- | --- |
| 1 | Acetaminophen |
| 2 | Aspirin |
| 3 | Celecoxib |
| 4 | Diclofenac |
| 5 | Diflunisal |
| 6 | Etodolac |
| 7 | Flurbiprofen |
| 8 | Ibuprofen |
| 9 | Indomethacin |
| 10 | Ketorolac |
| 11 | Meloxicam |
| 12 | Naproxen |
| 13 | Nimesulide |
| 14 | Oxaprozin |
| 15 | Piroxicam |
| 16 | Salsalate |
| 17 | Sulindac |

Table S2. Baseline characteristics of participants after propensity score matching.

| Variables | NSAIDs usage | | *p*-value | SMD |
| --- | --- | --- | --- | --- |
|  | No (n =1551) | Yes (n =1551) |  |  |
| Age (years), Mean ± SD | 59.8 ± 11.0 | 59.7 ± 10.5 | 0.782 | 0.010 |
| Male, n (%) | 657 (42.4) | 662 (42.7) | 0.856 | 0.007 |
| Race/ethnicity, n (%) |  |  | 0.839 | 0.043 |
| Mexican American | 234 (15.1) | 237 (15.3) |  |  |
| Other Hispanic | 146 (9.4) | 155 (10.0) |  |  |
| Non-Hispanic White | 731 (47.1) | 743 (47.9) |  |  |
| Non-Hispanic Black | 335 (21.6) | 324 (20.9) |  |  |
| Other Race | 105 (6.8) | 92 (5.9) |  |  |
| Level of education, n (%) |  |  | 0.480 | 0.025 |
| Did not graduate from high school | 455 (29.3) | 473 (30.5) |  |  |
| high school level or above | 1096 (70.7) | 1078 (69.5) |  |  |
| Marital status, n (%) |  |  | 0.827 | 0.008 |
| Living alone | 657 (42.4) | 651 (42.0) |  |  |
| Married or living with a partner | 894 (57.6) | 900 (58.0) |  |  |
| BMI, kg/m^2^, Mean ± SD | 31.6 ± 7.7 | 31.5 ± 7.7 | 0.706 | 0.008 |
| PIR (IQR) | 1.6 (1.0, 3.2) | 1.6 (0.9, 3.3) | 0.814 | 0.010 |
| Smoking status, n (%) |  |  | 0.801 | 0.024 |
| Never | 653 (42.1) | 643 (41.5) |  |  |
| Current | 377 (24.3) | 393 (25.3) |  |  |
| Past | 521 (33.6) | 515 (33.2) |  |  |
| Alcohol status, n (%) |  |  | 0.482 | 0.025 |
| No | 1072 (69.1) | 1090 (70.3) |  |  |
| Yes | 479 (30.9) | 461 (29.7) |  |  |
| Physical activity, n (%) |  |  | 0.825 | 0.022 |
| Sedentary | 681 (43.9) | 697 (44.9) |  |  |
| Moderate | 513 (33.1) | 499 (32.2) |  |  |
| Vigorous | 357 (23) | 355 (22.9) |  |  |
| Hypertension |  |  | 0.847 | 0.007 |
| No | 496 (32.0) | 491 (31.7) |  |  |
| Yes | 1055 (68.0) | 1060 (68.3) |  |  |
| Diabetes |  |  | 0.379 | 0.032 |
| No | 1031 (66.5) | 1054 (68.0) |  |  |
| Yes | 520 (33.5) | 497 (32.0) |  |  |
| eGFR, (mL/min/1.73 m²) | 85.5 ± 20.6 | 85.0 ± 20.3 | 0.498 | 0.024 |

Note: Abbreviation: BMI body mass index, PIR poverty income ratio, eGFR estimated glomerular filtration rate
